# Cholera in Oxford
*Memoir of the Cholera in Oxford in the Year 1854. By H. Wentworth Acland, M.D., F.R.S. London: Churchill. 1856.


**Published:** 1856-07

**Authors:** 


### CHOLERA IN OXFORD.*

Dr. Acland has presented to the public a very hand-

* Memoir of the Cholera in Oxford in the Year 1854. By H. Wentworth Acland, M.D., F.R.S. London: Churchill. 1856.

some classical and interesting work, which will deserve well to be referred to at greater length on another occasion.

The conclusions at which he arrives as to the causes of cholera, are:

Diarrhœa always co-exists with cholera in any given locality, and is not communicated from person to person.

Cholera may arise without the suspicion of contagion.

Cholera may certainly be conveyed from place to place by human agency.

It can scarcely be longer doubted that the evacuations of cholera patients are capable of communicating the cholera.

It is quite certain that in the majority of cases the cholera evacuations do not communicate the cholera.

It is quite certain that in localities apparently exceedingly prone to the development of cholera, cholera which is imported to them may not be propagated.

The poison of the evacuations (of cholera patients) may be conveyed through the air or by the agency of water; therefore poisoned water, though one means of spreading the disease, is not the only means.

Crowded dwellings and imperfect ventilation are dangerous in the highest degree during the prevalence of a cholera atmosphere.

A low scale of diet favours diarrhœa; a better diet tends to check it.

